# One-Step Synthesis of Al-Doped UiO-66 Nanoparticle for Enhanced Removal of Organic Dyes from Wastewater

**DOI:** 10.3390/molecules28052182

**Published:** 2023-02-26

**Authors:** Panpan Liu, Jiafei Lyu, Peng Bai

**Affiliations:** 1 Department of Pharmaceutical Engineering, School of Chemical Engineering and Technology, Tianjin University, Tianjin 300350, China; 2 Key Laboratory of Systems Bioengineering, Ministry of Education, Tianjin 300350, China

**Keywords:** metal−organic framework, Al-doped, adsorption, organic dyes, mechanism

## Abstract

In this study, a series of Al-doped metal-organic frameworks (Al_x_Zr_(1−x)_-UiO-66) were synthesized through a one-step solvothermal method. Various characterization techniques, including X-ray diffraction, X-ray photoelectron spectroscopy, Fourier transform infrared spectroscopy, and N_2_ sorption measurement, suggested that the Al doping was uniform and barely influenced the crystallinity, chemical stability, and thermal stability of the materials. Two cationic dyes, safranine T (ST) and methylene blue (MB), were selected for investigating the adsorption performances of Al-doped UiO-66 materials. Al_0.3_Zr_0.7_-UiO-66 exhibited 9.63 and 5.54 times higher adsorption capacities than UiO-66, 498 mg/g and 251 mg/g for ST and MB, respectively. The improved adsorption performance can be attributed to π-π interaction, hydrogen bond, and the coordination between the dye and Al-doped MOF. The pseudo-second-order and Langmuir models explained the adsorption process well, which indicated that the dye adsorption on Al_0.3_Zr_0.7_-UiO-66 mostly occurred through chemisorption on homogeneous surfaces. A thermodynamic study indicated the adsorption process was spontaneous and endothermic. The adsorption capacity did not decrease significantly after four cycles.

## 1. Introduction

Organic dyes are commonly used in a wide range of manufacturing industries, including textiles, printing, plastics, and paper, leading to a large amount of dye-containing wastewater [1,2,3]. ST and MB are common representatives of cationic dyes, which are mainly used as redox indicators and biological dyes, with complex composition, high toxicity, and are not easy to degrade. The discharge of these dyes into the environment will pose a serious threat to the ecosystem and human health. Effective treatment of organic dyes is critical instead of being discharged into the environment [4]. Among the dye removal methods, including adsorption, electrolysis, flocculation, and biodegradation [5,6,7,8], adsorption has been widely utilized due to its advantages in environmental friendliness, high efficiency, and low cost. Various adsorbents with robust stability and high surface area have been investigated to remove organic dye contaminants, such as activated carbon, borosilicate, and zeolite [9,10,11,12]. To date, a great deal of research has been conducted for novel adsorbents that can effectively remove the dye from water.

As porous materials with periodic networks, metal-organic frameworks (MOFs) are composed of metal nodes and organic ligands through coordination connections [13,14]. Due to their tailorable functionality, designable porosity, high surface area, and abundance of active sites [15,16,17,18], MOFs hold great potential for the adsorptive removal of pollutants from the water environment and have been extensively explored by many researchers to remove organic dye pollutants from water environments [4,19]. Haque et al. investigated MIL-101 (Cr) and MIL-53 (Cr) to prove the significance of porosity for the adsorptive removal of methyl orange (MO) [20]. Tehrani et al. observed excellent performance of MIL-68(Al) for efficient adsorption of methylene blue and rhodamine B [21].

UiO-66, constructed from Zr_6_ clusters [Zr_6_O_4_(OH)_4_] and terephthalate linkers [22,23], exhibited strong chemical and thermal stability [24,25,26] and excellent adsorption properties for the removal of pollutants in aqueous solutions such as dyes, antibiotics, and heavy metal ions [1,27,28]. In recent years, it has been found that metal doping can strengthen adsorption performance. Yang et al. reported Mn-doped UiO-66 with higher surface area and pore size with increased adsorption properties of tetracycline and Cr(IV) [24]. Goyal et al. reported enhanced water stability of Fe-doped HKUST-1 with improved Pb(II) adsorption performance [29].

In this study, Al has been chosen as the binding partner for UiO-66 to form metal-doped MOFs to further explore the adsorption potential of UiO-66. A series of Al-doped materials (Al_x_Zr_(1−x)_-UiO-66) were one-step synthesized and characterized by X-ray diffraction (XRD), X-ray photoelectron spectroscopy (XPS), Fourier transform infrared (FTIR), thermal gravimetric analysis (TGA), N_2_ sorption measurement for adsorptive removal of organic dyes from water where a detailed adsorption study was conducted on the optimal Al_0.3_Zr_0.7_-UiO-66 including the effects of pH, contact time, and initial dye concentrations. A plausible adsorption mechanism was proposed based on the dye adsorption performance, zeta potentials, XPS analysis, involving π-π interaction, hydrogen bond, and Al-N coordination bond between the dye and Al-doped UiO-66.

## 2. Results and Discussion

### 2.1. Characterization of As-Synthesized Adsorbents

**Figure 1:**
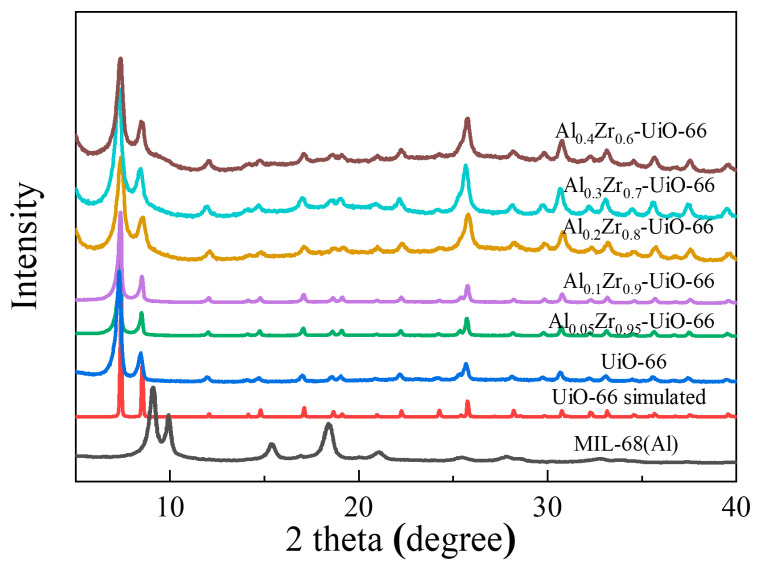
Comparison of the experimental and simulated XRD patterns.

The composition and crystallinity of the as-synthesized adsorbents could be characterized by powder X-ray diffraction (PXRD) (Figure 1), including Al_x_Zr_(1−x)_-UiO-66 (x = 0.05, 0.1, 0.2, 0.3, and 0.4) and pristine UiO-66. The characteristic peaks of prepared UiO-66 matched well with its simulated pattern, indicating a successful synthesis. As the Al precursor was included in the solvothermal synthesis, the principal diffraction peaks of Al_x_Zr_(1−x)_-UiO-66 were consistent with those of pristine UiO-66. Al_x_Zr_(1−x)_-UiO-66 MOFs exhibited slightly lower diffraction intensity though. No diffraction peaks were observed for aluminum or aluminum oxide species, which proved the absence of other Al species. At the same time, the characteristic diffraction of MIL-68(Al) was not observed, indicating the synthesis of phase-pure Al-doped UiO-66 instead of the physical mixture of UiO-66 and MIL-68(Al).

**Figure 2:**
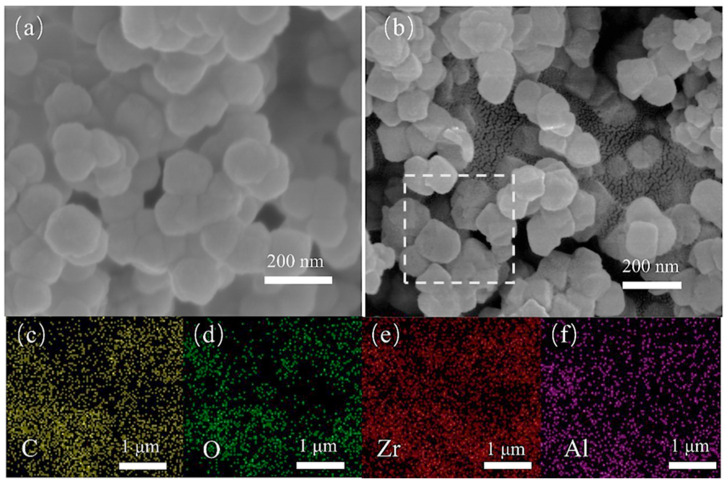
SEM images of UiO-66 (**a**) and Al_0.3_Zr_0.7_-UiO-66 (**b**); and EDS mappings of the portion selected: distribution of C (**c**), O (**d**), Zr (**e**), and Al (**f**).

The pristine UiO-66 exhibited an agglomerated morphology of ~160 nm nanocrystals, while the Al-doped UiO-66 sample exhibited a more uniform dispersion of ~115 nm particles (Figure 2a,b). The homogeneous distribution of Al in the framework was proved by EDS mapping (Figure 2c–f and Table S1). ICP analysis has been implemented to determine the actual amount of Al involved in the whole series of materials (Table S2). The actual amount of Al increased gradually with the increased initial doping amount in the synthesis process. XPS measurement further confirmed the presence of Al in the framework (Figure S1).

**Figure 3:**
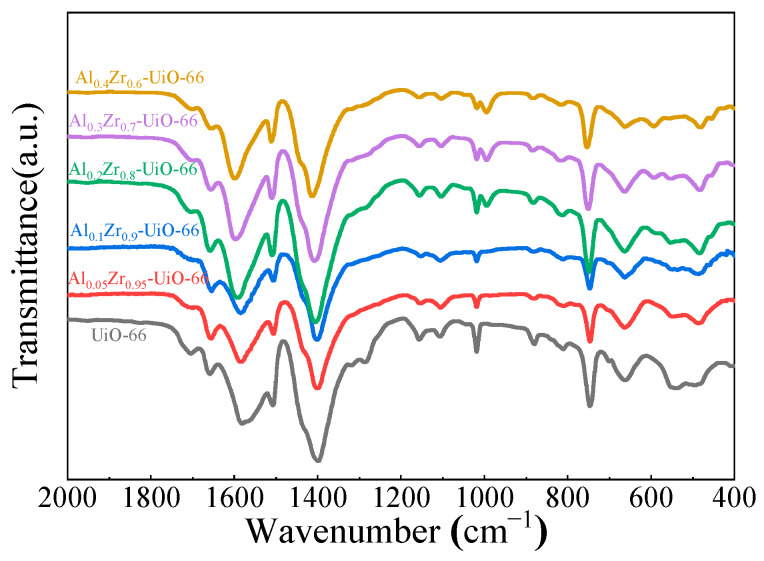
FTIR spectra of UiO-66 and Al-doped UiO-66 MOFs.

Similar FT-IR spectra of UiO-66 and Al_0.3_Zr_0.7_-UiO-66 were observed, as shown in Figure 3. The two obvious bands, approximately 1399 and 1585 cm^−1^, could be ascribed to the symmetric and asymmetric stretch vibrations of the O=C-O-group, as confirmed by their presence in the framework. The band of approximately 1100 cm^−1^ belonged to Zr-O vibration. The vibration bands, approximately 745 cm^−1^ and 666 cm^−1^, are characteristic bands of the O-H and C-H vibrations in the H_2_BDC ligand [24,30]. The peak at 590 cm^−1^ in Al_0.3_Zr_0.7_-UiO-66 could be assigned to the Al-O stretching vibration [31,32,33], which also indicates that Al was involved in the formation of Al_x_Zr_(1−x)_-UiO-66 MOF.

**Figure 4:**
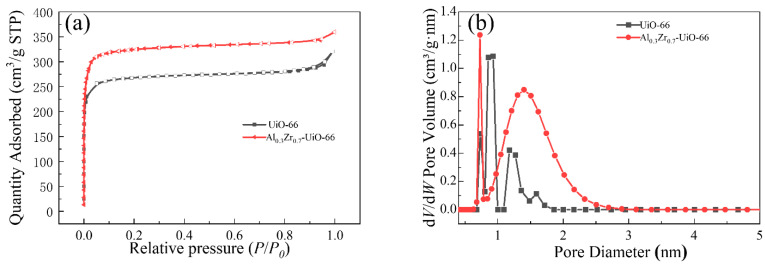
(**a**) N_2_ adsorption-desorption isotherms and (**b**) DFT-calculated pore size distributions of UiO-66 and Al_0.3_Zr_0.7_-UiO-66.

To determine the pore size, pore volume (*V_t_*), and surface area of the Al-doped material, N_2_ adsorption-desorption tests were performed on UiO-66 and Al_0.3_Zr_0.7_-UiO-66 at 77 K. As shown in Figure 4a, both UiO-66 and Al_0.3_Zr_0.7_-UiO-66 exhibited the typical type I isotherms, which reflected the typical microporous structure. Based on the Brunauer–Emmett–Teller (BET) method and density functional theory (DFT), the specific surface area and total pore volume of MOFs could be obtained, as listed in Table 1. After Al doping UiO-66, the Al_0.3_Zr_0.7_-UiO-66 sample attained a larger BET surface area and total pore volume with more active sites, which was expected to show a higher adsorption capacity than pristine UiO-66. It should be noted that the pore size distribution of Al-doped MOF material is larger than that of pristine UiO-66, which could be a potential for adsorption and separation of macromolecular organic dyes in water (Figure 4b).

**Figure 5:**
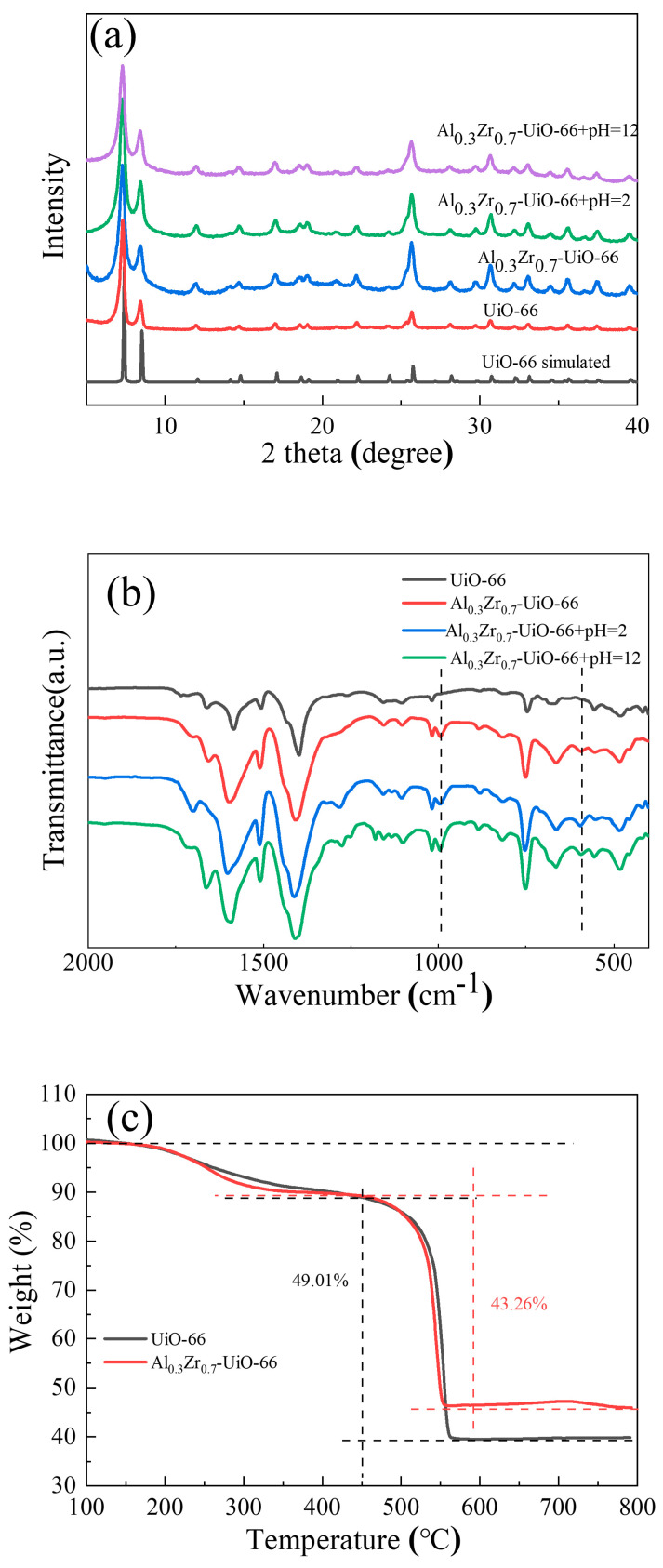
(**a**) XRD patterns, (**b**) FTIR spectra of Al_0.3_Zr_0.7_-UiO-66 before and after exposure to aqueous solutions at pH = 2 and pH = 12, and (**c**) TGA curves of UiO-66 and Al_0.3_Zr_0.7_-UiO-66.

For materials to be used in practical applications, they must exhibit excellent structural stability. According to relevant reports [34,35], the structural stability of MIL-68(Al) can only be maintained when the pH is between 3.5 and 8.0. Therefore, it is indispensable to study the chemical and thermal stability of Al-doped MOF. Taking the case of Al_0.3_Zr_0.7_-UiO-66, the samples were immersed in aqueous solutions of pH = 2 and pH = 12 for 24 h, followed by sample collection and analysis with XRD and FTIR, as shown in Figure 5a,b. The XRD patterns remained almost unchanged, and the characteristic peaks of Al-doped MOF were preserved in the FTIR spectra (Figure 5b), demonstrating that Al_0.3_Zr_0.7_-UiO-66 can maintain superior chemical stability between pH = 2 and 12. TGA analysis in the air atmosphere confirmed the thermal stability of Al-doped material, where three weight loss steps have been observed on UiO-66 and Al-doped UiO-66 in the temperature range of 40 ~ 800 °C (Figure 5c). The first step in weight loss within 200 °C originated from the removal of residual solvent and water in the structure. The second weight loss step in the temperature range of 200 ~ 400 °C was due to the dehydroxylation of the framework μ_3_-OH of the UiO-66. The weight loss after 450 °C was mainly due to the complete collapse of the structure caused by the combustion decomposition of ligands, which was confirmed by an obvious exothermic peak in the differential scanning calorimeter (DSC) curves (Figure S2) [36,37,38,39]. By analyzing the weight percentage of ligand and residual metal oxide solid, the amount of missing linker defect per Zr_6_ cluster of UiO-66 and Al_0.3_Zr_0.7_-UiO-66 was calculated to be 0.61 and 1.52, respectively. More defects resulted in a larger BET surface area and pore size distribution in Al_0.3_Zr_0.7_-UiO-66, which was conducive to improved dye adsorption performance.

### 2.2. Adsorption Performance

**Figure 6:**
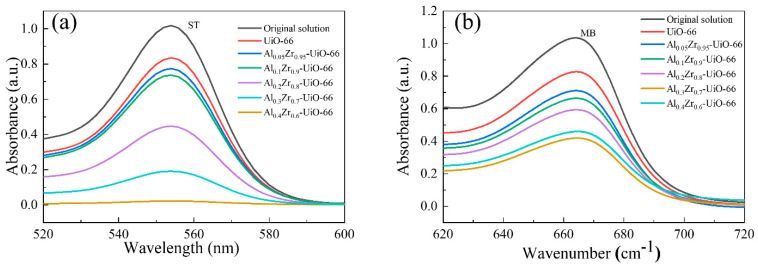
The UV-vis absorption spectra of two dyes (**a**) ST and (**b**) MB before and after adsorption on the pristine UiO-66 and Al_x_Zr_(1−x)_-UiO-66 MOFs (adsorption time: 12 h, initial dye concentration: 300 mg/L, adsorbent dosage: 1 g/L, adsorption temperature: 30 °C).

The adsorption performances of two common cationic dyes, MB and ST, doped with and without Al, were investigated on UiO-66 and Al-doped UiO-66. Figure 6 shows the UV-vis absorption spectra of two dyes (ST and MB) before and after 12 h of adsorption on the pristine UiO-66 and Al_x_Zr_(1−x)_-UiO-66 MOFs. Compared with the absorbance before adsorption, the absorbance change in two dyes after Al_x_Zr_(1−x)_-UiO-66 adsorption was greater than that of the pristine UiO-66, indicating that Al_x_Zr_(1−x)_-UiO-66 improved the adsorption performance, among which Al_0.3_Zr_0.7_-UiO-66 exhibited excellent performance, no matter for adsorption of ST or MB. The ST and MB adsorption capacities of Al_0.3_Zr_0.7_-UiO-66 were 287.29 mg/g and 183.76 mg/g, with a removal rate of 95.76% and 61.25%, respectively. In contrast, the removal rate of UiO-66 for ST and MB was as low as 13.04% and 12.12% (Table 2).

The above results indicated that Al-doped UiO-66 improved the adsorption performance compared with pristine UiO-66, which was mainly caused by the following reasons: The higher specific surface area of Al_0.3_Zr_0.7_-UiO-66 consequently generated more adsorption sites, which could be confirmed by the characterization results of N_2_ adsorption and desorption. It is difficult for ST and MB with bulky structures (Figure S3) to diffuse through the network of pristine UiO-66, while Al_0.3_Zr_0.7_-UiO-66 provided a larger pore size, allowing for fast diffusion of dye molecules. The zeta potential of the adsorbents was observed where surface positive charge reduced from 36.5 mV to 22.9 mV as Al was doped into UiO-66, leading to enhanced electrostatic interaction between Al_0.3_Zr_0.7_-UiO-66 and cationic dye molecules (Figure S4).

The adsorption performance of Al-doped UiO-66 for ST and MB was different to some extent. A larger ST adsorption capacity than MB in Al_0.3_Zr_0.7_-UiO-66 could be attributed to three reasons: (1) ST has a larger conjugate plane for strong π-π interaction; (2) the amino groups of ST could form hydrogen bonds with the hydroxyl groups in Al_0.3_Zr_0.7_-UiO-66; (3) the coordination between nitrogenous groups of ST and Al-doped UiO-66, which could be obtained from XPS analysis explained in detail in the adsorption mechanism section.

### 2.3. Adsorption Kinetics

**Figure 7:**
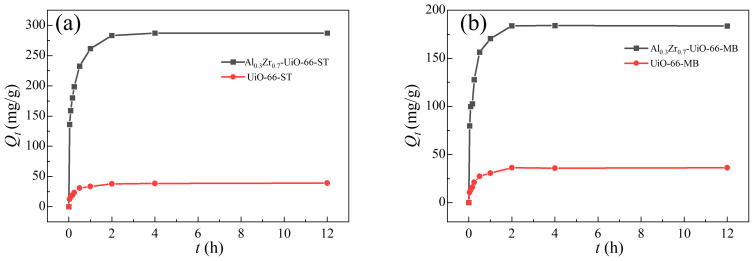
Adsorption kinetics of (**a**) ST and (**b**) MB in UiO-66 and Al_0.3_Zr_0.7_-UiO-66.

The adsorption kinetics could be determined by analyzing the adsorption capacity of adsorbents at various intervals of time during the adsorption process performed at 30 °C with an initial dye concentration of 300 mg/L (Figure 7 and Figure S5). Due to the abundance of adsorption sites at the beginning, the ST and MB adsorption amounts increased rapidly for the first hour. As the available adsorption sites of the two adsorbents gradually decreased, the adsorption tended to reach saturation, reaching the adsorption equilibrium within two hours.

Two adsorption kinetics models, the pseudo-second-order model and the intraparticle diffusion model, were employed to describe the adsorption process of two dyes over pristine UiO-66 and Al_0.3_Zr_0.7_-UiO-66 (Table 3). The correlation coefficients of the pseudo-second-order model, being very close to one, indicated a good fit, and the calculated equilibrium capacities were very close to the experimental results (Figure S6 and Table 3). Therefore, it could be determined that the chemisorption reaction dominated the adsorption reaction, involving the sharing or exchange of electrons between dye molecules and the adsorbent. Three stages were obtained by further fitting the intraparticle diffusion model (Figure S6). While the adsorption time gradually increased, the adsorption capacity of dyes increased sharply at first, and then the slope of the fitting line became very small until the final equilibrium was reached, which indicated that the diffusion and migration of dyes within the framework might be the limiting step.

### 2.4. Adsorption Isotherms

**Figure 8:**
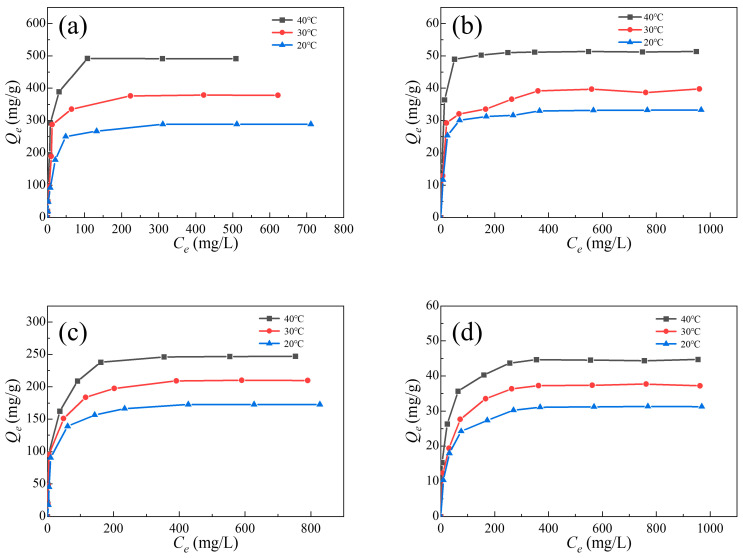
ST and MB adsorption isotherms in (**a**,**c**) Al_0.3_Zr_0.7_-UiO-66 and (**b**,**d**) UiO-66.

The adsorption isotherms of two dyes on pristine UiO-66 and Al_0.3_Zr_0.7_-UiO-66 were collected with different initial dye concentrations of 20, 50, 100, 200, 300, 400, 600, 800, and 1000 mg/L at 20, 30, and 40 °C (Figure 8). It could be observed that the equilibrium adsorption capacities increased with the rising dye concentration until the maximum adsorption capacities were achieved.

Adsorption isotherms of ST and MB on UiO-66 and Al_0.3_Zr_0.7_-UiO-66 were fitted using Langmuir, Freundlich, and Henry models (Figures S7 and S8, and Table 4). The Henry model and Freundlich model describe the adsorption isotherm with low *R^2^* values, while the Langmuir model fits the data well, indicating that the adsorption of dyes on UiO-66 and Al_0.3_Zr_0.7_-UiO-66 could be a monolayer on the uniform surface.

The maximum theoretical adsorption capacities of ST and MB on Al_0.3_Zr_0.7_-UiO-66 calculated with the Langmuir model are 497.51 mg/g and 251.26 mg/g, approximately 9.63 times and 5.54 times that of UiO-66, respectively. Compared with results in the literature (Table S3), it was found that the Al_0.3_Zr_0.7_-UiO-66 showed significantly larger performance for ST and MB adsorption in an aqueous solution.

The adsorption properties of UiO-66 and Al_0.3_Zr_0.7_-UiO-66 could be obtained by relating the performance index PC to the initial concentration, as shown in Figure S9. When the initial concentration of the dyes increased, the active sites on the surface of the adsorbent samples might be saturated, leading to a reduction in PC values. However, at the same initial concentration, the PC value of Al_0.3_Zr_0.7_-UiO-66 was greater than that of UiO-66, indicating the better adsorption performance of Al_0.3_Zr_0.7_-UiO-66 than that of UiO-66.

### 2.5. Adsorption Thermodynamics

In order to further understand the adsorption behavior, thermodynamic studies were carried out. The thermodynamic parameters of organic dyes over Al_0.3_Zr_0.7_-UiO-66, including Gibbs free energy change (*ΔG*), enthalpy change (*ΔH*), and entropy change (*ΔS*), were obtained by applying the following formula:

ln*K_A_* = *ΔS*/*R* − *ΔH*/*RT*, (1)

**Figure 9:**
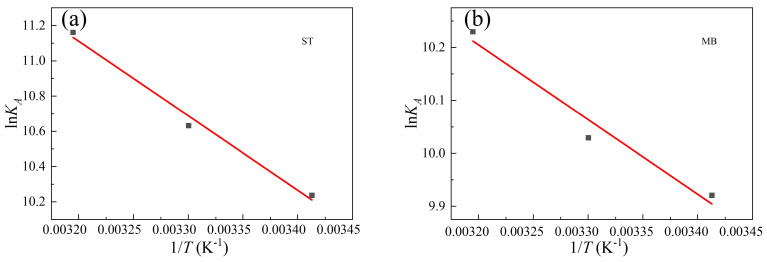
Van’t Hoff plot of (**a**) ST adsorption and (**b**) MB adsorption.

*ΔG* = −*RT*ln*K_A_* (2)

where *R* is the universal gas constant, equal to 8.314 J·mol^−1·^K^−1^; *T* is the Kelvin temperature (K), *K_A_* is the adsorption constant obtained through Langmuir model fitting, which is the best fitting model for adsorption isothermal data. As could be seen from Figure 9 and Table 5, the thermodynamic parameters including *ΔG*, *ΔH*, and *ΔS* were calculated, where a negative *ΔG* value demonstrated a spontaneous adsorption process and a positive *ΔH* indicated an endothermic adsorption process. The positive *ΔS* value suggested increased disorder at the adsorbent-adsorbate interface.

### 2.6. Regeneration and Reusability

**Figure 10:**
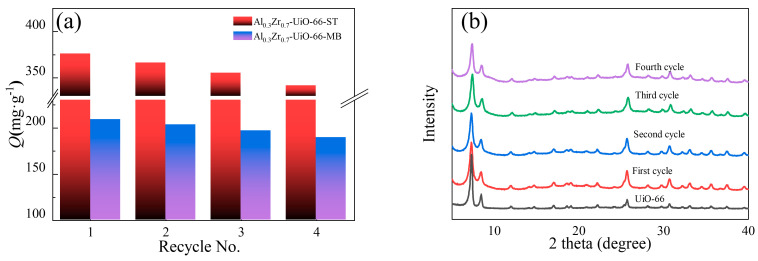
(**a**) Four-cycle ST and MB adsorption on Al_0.3_Zr_0.7_-UiO-66; (**b**) XRD patterns of Al_0.3_Zr_0.7_-UiO-66 after each cycle.

After four cycles of adsorption and desorption, the crystal structure of Al-doped UiO-66 was preserved, which was confirmed by XRD (Figure 10b). Figure 10a shows the ST and MB adsorption capacities over Al-doped UiO-66 and regenerated Al-doped UiO-66. It was found that the regeneration efficiency was significant, and the adsorption capacity decreased by less than 10% after four adsorption cycles, which indicated the potential of the Al-doped UiO-66 in commercial adsorptions.

### 2.7. Mechanism of Adsorption

According to Figure S10, ST and MB FTIR bands were observed after adsorption on Al_0.3_Zr_0.7_-UiO-66, which indicated that ST and MB had successfully transferred from aqueous solution to Al_0.3_Zr_0.7_-UiO-66. When Al was doped into UiO-66, the decrease in surface positive charge resulted in enhanced electrostatic interaction between Al_0.3_Zr_0.7_-UiO-66 and cationic dye molecules. However, the stable adsorption capacity at different pH values indicated that electrostatic interaction was not the main factor determining the adsorption performance of Al_0.3_Zr_0.7_-UiO-66 (Figure S11).

**Figure 11:**
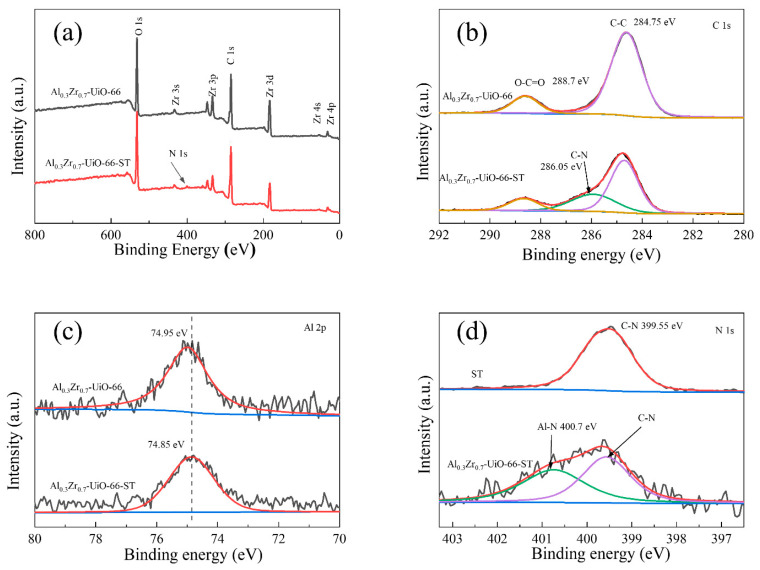
XPS analysis of Al_0.3_Zr_0.7_-UiO-66 before and after adsorption of ST: (**a**) XPS spectra, (**b**) XPS spectra of C 1s, (**c**) XPS spectra of Al2p, and (**d**) N1s spectra of ST and Al_0.3_Zr_0.7_-UiO-66 after the adsorption of ST.

In order to further elucidate the surface interactions between Al_0.3_Zr_0.7_-UiO-66 and dye molecules, XPS analysis was performed to determine the elemental states. The N1s peak appeared in the XPS spectra of Al_0.3_Zr_0.7_-UiO-66 after ST adsorption, indicating that dye molecules were successfully adsorbed on Al_0.3_Zr_0.7_-UiO-66 (Figure 11a). The peak emerged at 286.05 eV in the C1s spectra, which corresponded to C–N (Figure 11b), which can be confirmed by the N 1s spectrum of ST (Figure 11d), further indicating that the dye molecule was concentrated on Al_0.3_Zr_0.7_-UiO-66 [40]. At the same time, the Al 2p peak shifted from 74.95 eV to 74.85 eV (Figure 11c), indicating the chemical interaction between Al and ST [33,41,42,43]. In comparison to ST, the N1s spectrum of Al_0.3_Zr_0.7_-UiO-66 after ST adsorption (Figure 11d) can be deconvoluted into two peaks, including the peak at 399.55 eV corresponding to the signals of the C−N from the structure of ST and the other peak at 400.7 eV, which was attributed to the Al-N coordination bond between the amino groups of ST and the Al sites in the network [33,42], indicating the potential coordinative connection in the process of ST adsorption on Al_0.3_Zr_0.7_-UiO-66.

The adsorption process of ST and MB on Al_0.3_Zr_0.7_-UiO-66 was complex, where organic dye molecules were first transferred from the solution to the outer surface of Al_0.3_Zr_0.7_-UiO-66, then diffused to the inner pore structure, and finally adsorbed by Al_0.3_Zr_0.7_-UiO-66. A larger BET surface area and pore size were observed for Al-doped UiO-66, which facilitated the adsorption. The weakening of electrostatic repulsion might also play a positive role in promoting adsorption. The π-π interaction between benzene rings in Al-doped MOF and the aromatic backbone of dyes could be also involved in the adsorption behavior. For ST adsorption, in addition to the aforementioned mechanism, hydrogen bonding between the amino group of ST and the hydroxyl group of Al-doped MOF and the coordination between Al and the nitrogen-containing group of ST could play a role. Therefore, the possible mechanism of the two dyes’, ST and MB, adsorption on Al-doped UiO-66 is shown in Figure S12.

## 3. Materials and Methods

### 3.1. Materials

All reactants and solvents used in this paper were of analytical-grade and without further purification. Terephthalic acid (H_2_BDC, 99.0%) and aluminum chloride hexahydrate (AlCl_3_·6H_2_O, 99.0%) were purchased from Shanghai Macklin Biochemical Technology Co., Ltd. (Shanghai, China), Zirconium chloride (ZrCl_4_, 99.9%) was supplied by Aladdin Reagent Company. These three reagents are used to synthesize samples. Ethanol and *N,N*-dimethylformamide (DMF) were purchased from Damao chemical reagent factory and used as washing agents and solvents. The organic dyes, including safranine T (ST) and methylene blue (MB), were used for the preparation of different concentrations of aqueous solutions. This study used deionized water for all of its experiments.

### 3.2. Synthesis of the Adsorbent

Al_x_Zr_(1−x)_-UiO-66 series with different Al: (Zr + Al) mole ratios were synthesized by a one-step hydrothermal method. For example, the polytetrafluoroethylene liner (50 mL) was loaded with ZrCl_4_ (0.163 g, 0.7 mmol), Al_3_Cl·6H_2_O (0.0724 g, 0.3 mmol), and DMF (20 mL), and ultrasound was performed for twenty minutes, followed by the addition of terephthalic acid (0.166 g, 1.0 mmol) to the liner. Ultrasonic stirring continued until all reactants were fully dissolved, then the mixture was heated at 120 °C for 24 h. In order to remove the unreacted materials from the as-synthesized products (denoted as Al_x_Zr_(1−x)_-UiO-66). Three rounds of washing with DMF and ethanol were performed. Five Al-doped MOF materials were prepared in this study, x = 0.05, 0.1, 0.2, 0.3, and 0.4, respectively, and the synthesis process of Al-doped UiO-66 is shown in Scheme S1. For comparison, the UiO-66 was also prepared in the absence of AlCl_3_·6H_2_O according to the same procedures, with the amount of ZrCl_4_ being 0.233 g (1.0 mmol).

### 3.3. Characterization

X-ray diffraction (PXRD) patterns of pristine UiO-66 and Al_x_Zr_(1−x)_-UiO-66 MOFs were obtained by a D/MAX-2500 X-ray Powder Diffractometer with Cu Kα radiation and a wavelength of 1.54178 Å in a scanning range of 5–40°. N_2_ adsorption–desorption measurements were conducted by a Micromeritics ASAP2460 surface area analyzer at 77 K for characterizing the pore size and specific surface area of pristine UiO-66 and Al_x_Zr_(1−x)_-UiO-66 MOFs. An STA449F5 (NETZSCH) thermogravimetric analyzer used air for thermal gravimetric analysis (TGA) at 10 °C/min. The dimensions and morphologies of samples were obtained using a Hitachi S-4800 scanning electron microscope (SEM). The elements were analyzed with energy dispersive spectroscopy (EDS) connected to SEM. X-ray photoelectron spectroscopy (XPS) analysis was conducted on a PHI 1600 ESCA instrument from PE Company equipped with an Al Kα X-ray radiation. The Fourier transform infrared (FT-IR) was conducted with a Nicolet 6700 FTIR spectrophotometer. A tablet machine was used for preparing KBr powder and the product into thin slices, and the spectra were recorded at 4000–400 cm^−1^. Zeta potentials were determined on the Mastersizer 3000 of Malvern Instruments. The pH meter was used to measure the pH value.

### 3.4. Adsorption Experiments, Kinetics, and Equilibrium Studies

The adsorption properties of two cationic dyes (ST and MB) over UiO-66 and Al_x_Zr_(1−x)_-UiO-66 were investigated. The dye concentrations before and after the adsorption experiment were determined by using a UV-vis spectrometer at their maximum absorbance values (λ_max-ST_ = 554 nm, λ_max-MB_ = 664 nm). Before adsorption, the adsorbents were dried in a vacuum oven at 100 °C for 10 h to remove the guest molecules in the pores. A typical adsorption experiment involved placing an exact amount of adsorbent (20.0 mg) into a specific concentration of dye solution (20 mL). The mixture was placed in an incubator and shaken at a specific temperature for 12 h. Then, the adsorbent was separated from the solutions by centrifugation, and the concentration of organic dyes in the supernatant was determined by the UV-vis spectrometer (UV-2100, Beijing Ruili Analytical Instrument Co., Ltd., Beijing, China). For evaluating the effect of pH, we adjusted the pH values using 1 M HCl and 1 M NaOH during the adsorption process. All of the adsorption experiments were performed at least three times, and the average value was calculated to obtain the adsorption amount.

The equilibrium adsorption capacityof dyes on the adsorbent was calculated by the following equation:

*Q_e_* = (*C_o_* − *C_e_*)*V*/*M*, (3)

where *Q_e_* (mg∙g^−1^) is the adsorption capacity at equilibrium, *C_o_* (mg∙L^−1^) is the initial concentration of the organic dye solution, *C_e_* (mg∙L^−1^) is the equilibrium concentration of the dye solution, *M* (g) is the mass of the adsorbent, and *V* (L) is the volume of the dye solution.

Two kinetic models were used to explain the kinetic result, including the pseudo-second-order model and intraparticle diffusion model [44,45]. The linear shapes of intra-particle diffusion and pseudo-second-order kinetic models are, respectively, expressed as follows:

*Q_t_* = *k_i_t*^0.5^ + *K*, (4)

*t*/*Q_t_* = 1/(*k*_2_*Q_e_*^2^) + *t*/*Q_e_*, (5)

where *Q_t_* and *Q_e_* (mg·g^−1^) represent the adsorption capacity at time *t* (h) and at equilibrium, respectively; *K* is a constant that is related to the boundary layer thickness. When the *K* value is equal to 0, that is, when the curve of *Q_t_* relative to *t*^0.5^ is a straight line through the origin, the adsorption process is affected only by the internal diffusion of particles; *k_i_* (mg·g^−1^·h^−0.5^) and *k*_2_ (g·mg^−1^·h^−1^) are the rate constants of intraparticle diffusion and pseudo-second-order, respectively;

Three models, including Langmuir, Henry, and Freundlich isotherms, were used to determine the adsorption mechanism and theoretical maximum adsorption capacity [46]. According to the Langmuir model, monolayer adsorption takes place on surfaces with a finite number of active sites. The Freundlich adsorption model was used to describe the adsorption characteristics of heterogeneous surfaces. The Henry model is an ideal model that reflects the linear relationship between dye concentration and adsorption capacity. The linear forms of the three models are as follows:

*C_e_*/*Q_e_* = 1/(*k_A_Q_m_*) + *C_e_*/*Q_m_*, (6)

log*Q_e_* = log*K_F_* + log*C_e_*/*n*, (7)

*Qe* = *K_p_C_e_*, (8)

where *Q_m_* (mg·g^−1^) is the theoretical maximum adsorption capacity of the adsorbent when the dye molecules form a uniform monolayer on the surface of the adsorbent; *K_A_* (L·mg^−1^) is the Langmuir adsorption equilibrium constant, which is related to the free adsorption energy. *K_F_* (mg/g(L/mg)^1/n^) is the Freundlich constant, and 1/n (unitless) represents the measure of adsorption intensity. *K_P_* (mL·g^−1^) represents the distribution constant.

For a better understanding, the adsorption performances of different adsorbents for various organic dyes in aqueous solutions, it is extremely important to determine their actual adsorption properties. Therefore, the partition coefficients (PC) of pristine UiO-66 and Al_0.3_Zr_0.7_-UiO-66 for two dyes were obtained in this work. The calculation formula for PC is as follows [1,47]:

PC = *Q_e_*/*C_e_*, (9)

where *Q_e_* (mg/g) and *C_e_* (mg/L) are the adsorption amount and concentration of dyes at adsorption equilibrium, respectively.

### 3.5. Regeneration and Recycling

In practical applications, the reproducibility and recyclability of adsorbents are extremely paramount. Therefore, to test the stability of MOF, regeneration and reuse research was carried out. After ST or MB adsorption, Al-doped UiO-66 was soaked in a sodium hydroxide solution and then washed with deionized water and anhydrous ethanol several times, then activated by vacuum heating at 100 °C for 12 h. After regeneration, XRD was performed on the samples, which were then reused for the next cycle of adsorption and desorption.

## 4. Conclusions

In this research, we successfully synthesized a stable Al-doped UiO-66 MOF material through the one-step solvothermal method. By adjusting the ratio of Al and Zr, it was found that Al_0.3_Zr_0.7_-UiO-66 exhibited the best adsorption performance. The adsorption capacities of Al_0.3_Zr_0.7_-UiO-66 for ST and MB were 9.63 and 5.54 times that of pristine UiO-66, respectively, at 313 K. Adsorption kinetics and isotherm studies demonstrated that the adsorption was a chemical adsorption process on a uniform surface. Adsorption thermodynamics research showed that the adsorption of ST and MB on Al_0.3_Zr_0.7_-UiO-66 was spontaneous and endothermic. A possible mechanism for improved adsorption performance was proposed. Compared with UiO-66, the weakened electrostatic repulsion of Al_0.3_Zr_0.7_-UiO-66 might have a positive effect on promoting adsorption. At the same time, the expansion of the pore size of Al-doped materials was also conducive to adsorption. The adsorption capacity of ST increased more than that of MB, possibly due to the influence of hydrogen bonding and coordination interactions between ST and Al_0.3_Zr_0.7_-UiO-66. Regeneration studies suggested that Al-doped MOF could be reused several times and maintain good structural integrity and high adsorption performance. Consequently, these results could provide a valuable strategy for developing efficient and stable MOF-based adsorbents to remove pollutants from water.

## Figures and Tables

**Table 1:** Textural properties of UiO-66 and Al_0.3_Zr_0.7_-UiO-66.

Sample	BET Area (m^2^/g)	Total Pore Volume (cm^3^/g)
UiO-66	849	0.50
Al_0.3_Zr_0.7_-UiO-66	1060	0.55

**Table 2:** The adsorption amount and removal rate of the two cationic dyes on UiO-66 and Al_x_Zr_(1−x)_-UiO-66 MOFs.

Samples	ST		MB	
	Adsorption Capacity (mg/g)	Removal Rate (%)	Adsorption Capacity (mg/g)	Removal Rate (%)
UiO-66	39.17	13.04	36.35	12.12
Al_0.05_Zr_0.95_-UiO-66	59.13	19.71	75.33	25.11
Al_0.1_Zr_0.9_-UiO-66	72.66	24.22	109.67	36.56
Al_0.2_Zr_0.8_-UiO-66	149.19	49.73	128.37	42.79
Al_0.3_Zr_0.7_-UiO-66	287.29	95.76	183.76	61.25
Al_0.4_Zr_0.6_-UiO-66	233.50	77.83	170.43	56.81

**Table 3:** Kinetic parameters of the pseudo-second-order and intraparticle diffusion models for ST and MB adsorption.

Adsorbents	Dyes	Pseudo-Second-Order Model				Intraparticle Diffusion Model		
		*Q_e,exp_* (mg·g^−1^)	*Q_e,cal_* (mg·g^−1^)	*K_2_* (g·mg^−1^·h^−1^)	*R^2^*	*K*	*ki*	*R^2^*
UiO-66	ST	39.17	39.56	0.2181	0.9998	23.85	9.95	0.9969
	MB	36.35	36.79	0.2148	0.9996	18.34	12.56	0.9957
Al_0.3_Zr_0.7_-UiO-66	ST	287.29	289.02	0.04912	0.9999	185.87	70.37	0.9380
	MB	183.76	184.84	0.08232	0.9998	130.27	38.34	0.9752

**Table 4:** Parameters of two isotherm models for ST and MB adsorption on UiO-66 and Al_0.3_Zr_0.7_-UiO-66.

Adsorbents	T (°C)	Dyes	Langmuir Model			Freundlich Model		
			*Q_m_* (mg·g^−1^)	*K_A_* (L·g^−1^)	*R^2^*	1/*n*	*K_F_*	*R^2^*
UiO-66	40 °C	ST	51.68	199.55	0.9999	0.1752	18.18	0.6506
		MB	45.37	73.51	0.9998	0.1944	13.55	0.8799
	30 °C	ST	40.23	56.47	0.9990	0.1838	12.63	0.7266
		MB	38.45	47.37	0.9994	0.2311	8.94	0.8927
	20 °C	ST	33.72	85.06	0.9998	0.1750	11.44	0.6635
		MB	32.18	45.44	0.9997	0.2270	7.66	0.8600
Al_0.3_Zr_0.7_-UiO-66	40 °C	ST	497.51	200.40	0.9999	0.3822	75.73	0.7788
		MB	251.26	86.65	0.9996	0.3228	38.93	0.8174
	30 °C	ST	384.62	118.07	0.9998	0.3738	52.34	0.8235
		MB	214.13	70.92	0.9996	0.3183	32.86	0.7784
	20 °C	ST	294.99	79.56	0.9999	0.3780	35.40	0.8308
		MB	176.68	63.61	0.9997	0.3283	25.26	0.7606

**Table 5:** Thermodynamic parameters for the adsorption of ST and MB over Al_0.3_Zr_0.7_-UiO-66.

Adsorbate	*T* (K)	*ΔG* (kJ·mol^−1^)	*ΔS* (J·mol^−1^·K^−1^)	*ΔH* (kJ·mol^−1^)
ST	293	−24.94	204.85	35.15
	303	−26.78		
	313	−29.04		
MB	293	−24.17	122.42	11.74
	303	−25.27		
	313	−26.62		

## Data Availability

The data are contained within the article.

## Supplementary Materials

The following supporting information can be downloaded at: https://www.mdpi.com/article/10.3390/molecules28052182/s1, Figure S1: XPS analysis of Al_0.3_Zr_0.7_-UiO-66: (a) XPS spectrum, (b) XPS spectrum of O 1s, (c) XPS spectrum of Al 2p; FigureS2: DSC curves of (a) UiO-66 and (b) Al_0.3_Zr_0.7_-UiO-66; Figure S3: Molecular structures of (a) ST and (b) MB; Figure S4: Zeta potential of UiO-66 and Al_0.3_Zr_0.7_-UiO-66 at the neutral pH; Figure S5: The UV-vis absorption spectra of ST or MB solution adsorption over (b, e) Al_0.3_Zr_0.7_-UiO-66 and (c, f) UiO-66 at different time intervals; Figure S6: The (a, b) pseudo-second-order kinetics plots and (c, d) intraparticle diffusion kinetics plots fittings of ST and MB adsorption on UiO-66 and Al_0.3_Zr_0.7_-UiO-66; Figure S7: The (a, b) Langmuir model and (c, d) Freundlich model isotherm fittings for ST adsorption on Al_0.3_Zr_0.7_-UiO-66 and UiO-66; Figure S8: The (a, b) Langmuir model and (c, d) Freundlich model isotherm fittings for MB adsorption on Al_0.3_Zr_0.7_-UiO-66 and UiO-66; Figure S9: The calculated PC of UiO-66 and Al_0.3_Zr_0.7_-UiO-66 as a function of initial dye concentrations. (a) ST, (b) MB dyes; Figure S10: FTIR spectra of Al_0.3_Zr_0.7_-UiO-66 before and after adsorption; Figure S11: The adsorption amount of Al_0.3_Zr_0.7_-UiO-66 on (a) ST and (b) MB varied with pHs; Figure S12: Schematic diagram of the proposed adsorption mechanism of two dyes’, ST and MB, adsorption on Al-doped UiO-66. Table S1: EDS analysis result of Al_0.3_Zr_0.7_-UiO-66; Table S2: ICP data of Al_x_Zr_(1-x)_-UiO-66 samples.; Table S3: Comparison of organic dye adsorption capacities of Al_0.3_Zr_0.7_-UiO-66 among various adsorbents [48,49,50,51,52,53,54,55,56,57,58,59,60,61,62,63,64,65,66,67,68,69,70].
