# Supplementary material for: One-Step Synthesis of Al-Doped UiO-66 Nanoparticle for Enhanced Removal of Organic Dyes from Wastewater
Source: Molecules. 2023 Feb 26;28(5):2182. doi: 10.3390/molecules28052182 (PMC10004798; doi:10.3390/molecules28052182)
Supplement: Supplementary file 1 [file molecules-28-02182-s001.zip › molecules-2197970-supplementary.pdf]

Supporting Information

One-Step Synthesis of Al-Doped UiO-66 Nanoparticle for Enhanced Removal of Organic Dyes fromWastewater

Panpan Liu [1,2], Jiafei Lyu [1,2,*] and Peng Bai [1,2,*]

1 Department of Pharmaceutical Engineering, School of Chemical Engineering and Technology, Tianjin University, Tianjin 300350, China

2 Key Laboratory of Systems Bioengineering, Ministry of Education, Tianjin 300350, China

* Correspondence: jflv@tju.edu.cn (J.L.); baipenga525@tju.edu.cn (P.B.)

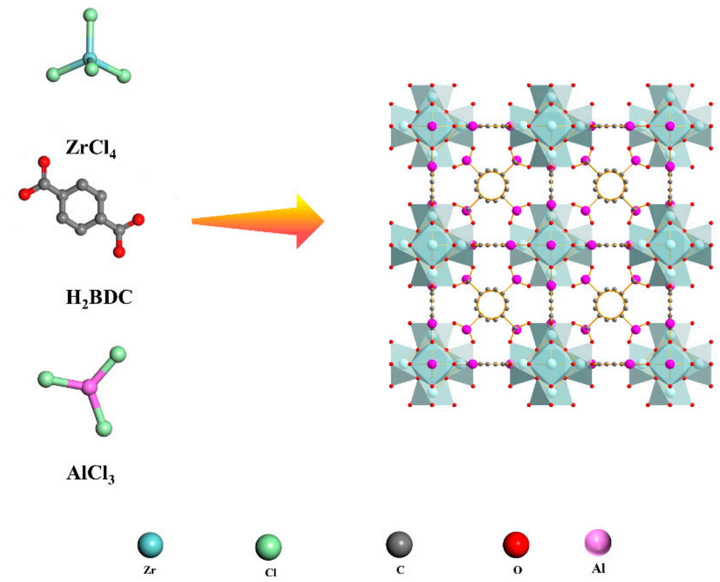

Scheme S1. The synthesis process of Al-doped UiO-66.

Table S1. EDS analysis result of $Al_{0.3}Zr_{0.7}$-UiO-66.

Element	Weight percentage (%)	Atomic percent (%)
C	53.17	74.07
O	19.49	20.38
Zr	26.10	4.79
Al	1.23	0.76

Table S2. ICP data of a series of Al-doped UiO-66 samples.

Sample	Al content wt%	Zr content wt%	Al/Zr molar ratio in the framework	Al/Zr molar ratio in precursor
$Al_{0.05}Zr_{0.95}$-UiO-66	0.50	25.64	0.066	0.053
$Al_{0.1}Zr_{0.9}$-UiO-66	0.97	26.35	0.13	0.11
$Al_{0.2}Zr_{0.8}$-UiO-66	1.86	25.03	0.25	0.25
$Al_{0.3}Zr_{0.7}$-UiO-66	2.54	15.82	0.54	0.43

$Al_{0.4}Zr_{0.6}$-UiO-66	3.35	16.27	0.70	0.67

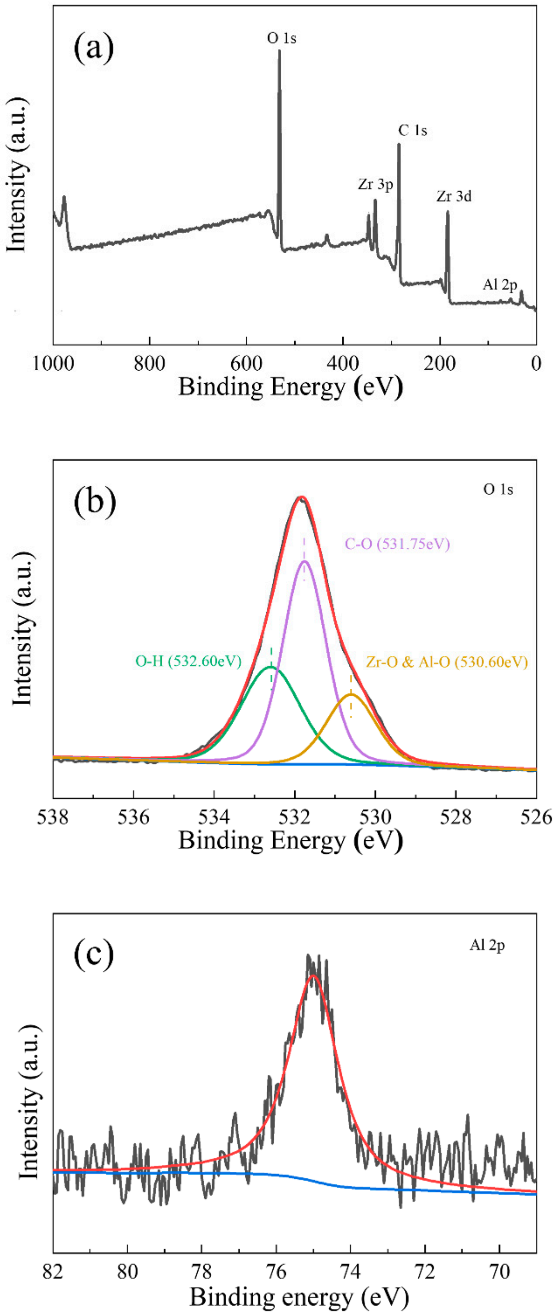

Figure S1. XPS analysis of $Al_{0.3}Zr_{0.7}$-UiO-66: (a) XPS spectrum, (b) XPS spectrum of O 1s, (c) XPS spectrum of Al 2p.

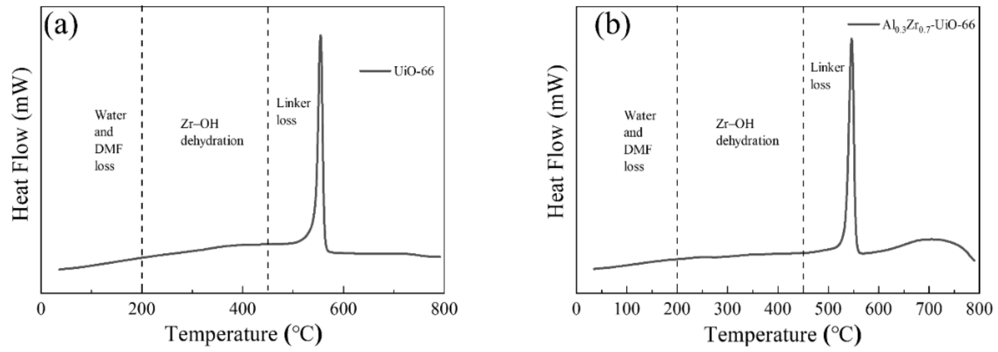

Figure S2. DSC curves of (a) UiO-66 and (b) $Al_{0.3}Zr_{0.7}$-UiO-66

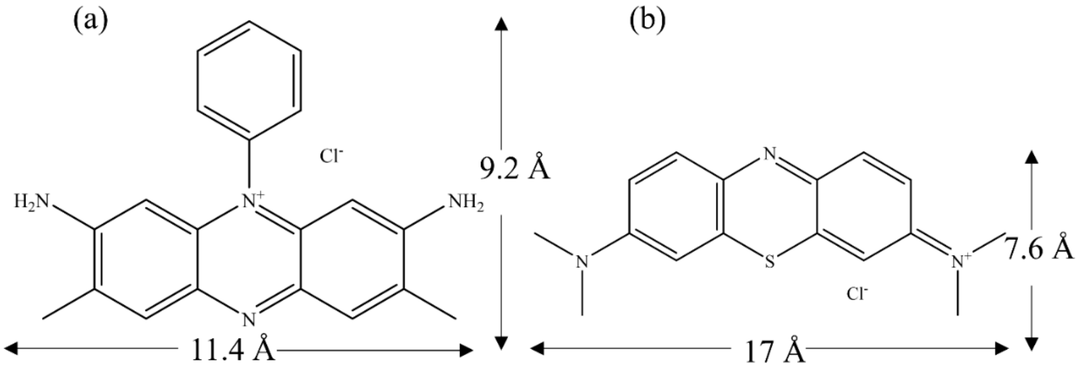

Figure S3. Molecular structures of (a) ST and (b) MB.

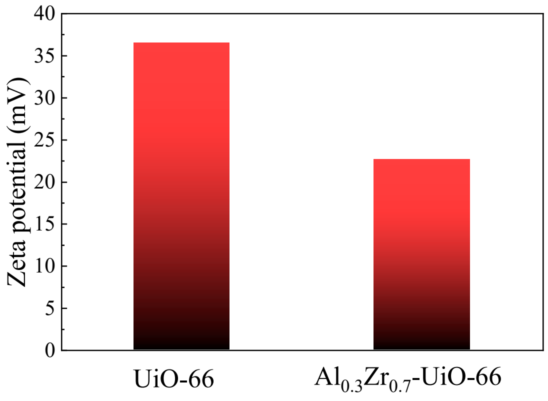

Figure S4. Zeta potential of UiO-66 and $Al_{0.3}Zr_{0.7}$-UiO-66 at the neutral *p*H.

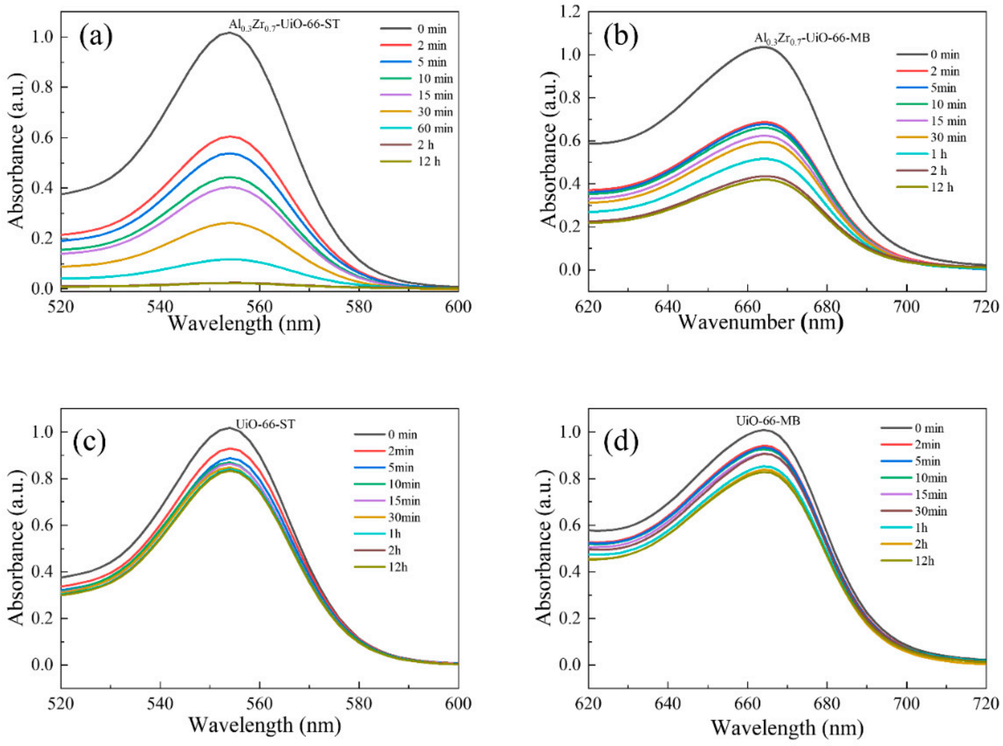

Figure S5. The UV-vis absorption spectra of ST or MB solution adsorption over (b, e) $Al_{0.3}Zr_{0.7}$-UiO-66 and (c, f) UiO-66 at different time intervals.

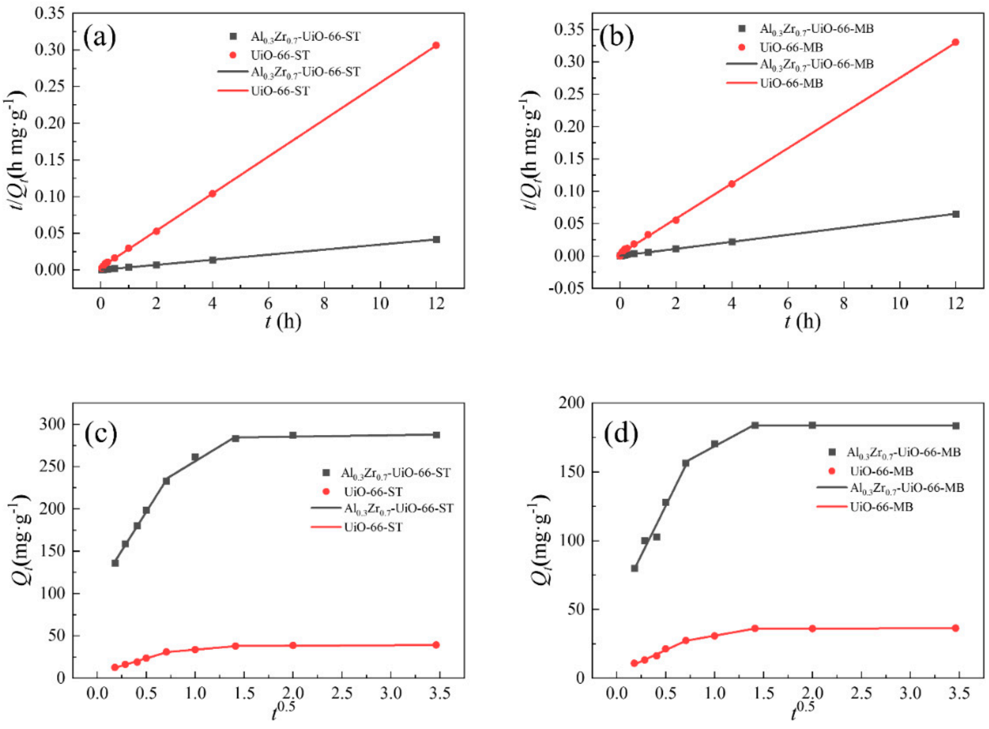

Figure S6. The (a, b) pseudo-second-order kinetics plots and (c, d) intraparticle diffusion kinetics plots fittings of ST and MB adsorption on UiO-66 and $Al_{0.3}Zr_{0.7}$-UiO-66.

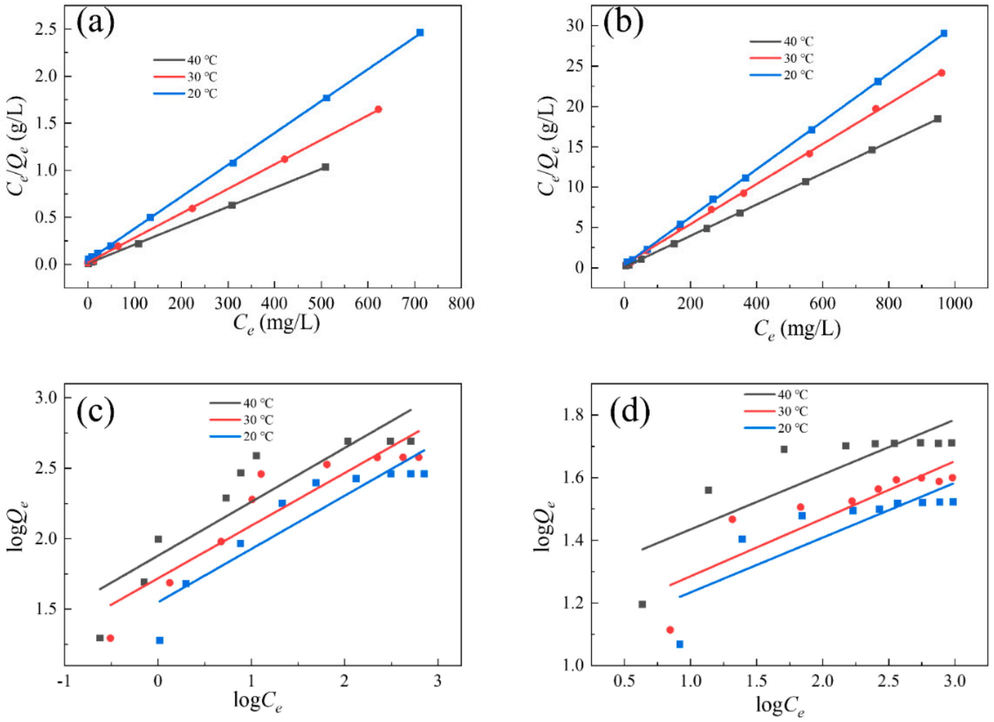

Figure S7. The (a, b) Langmuir model and (c, d) Freundlich model isotherm fittings for ST adsorption on $Al_{0.3}Zr_{0.7}$-UiO-66 and UiO-66.

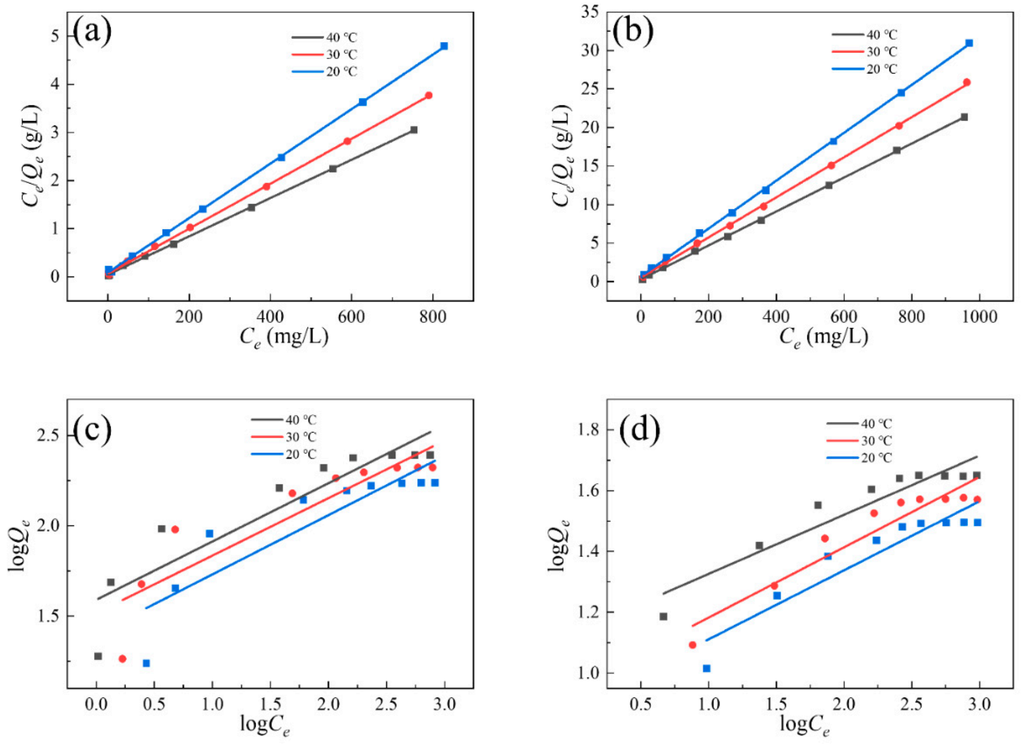

Figure S8. The (a, b) Langmuir model and (c, d) Freundlich model isotherm fittings for MB adsorption on $Al_{0.3}Zr_{0.7}$-UiO-66 and UiO-66.

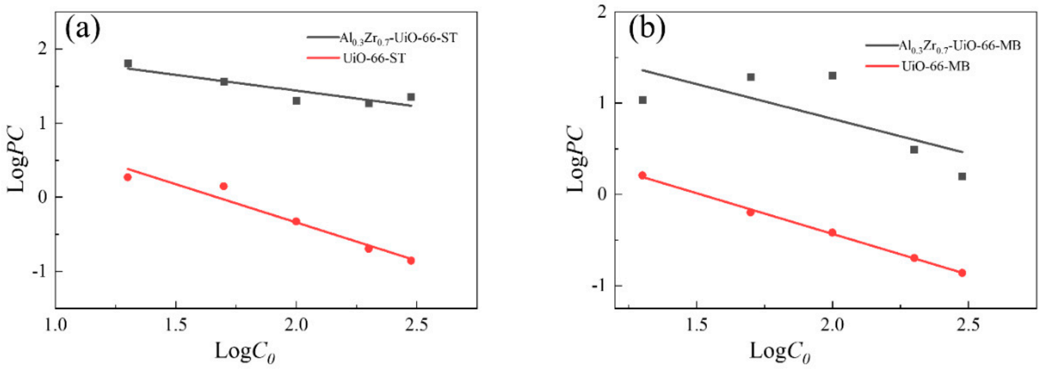

Figure S9. The calculated PC of UiO-66 and $Al_{0.3}Zr_{0.7}$-UiO-66 as a function of initial dye concentrations. (a) ST, (b) MB dyes.

Table S3. Comparison of organic dyes adsorption capacities of $Al_{0.3}Zr_{0.7}$-UiO-66 among various adsorbents.

Adsorbate	Adsorbents	*T* (K)	Q_e(mg·g^{-1})	refs
ST	MR-FeS@SC	303	102.14	[48]
	MCM-41	303	68.8	[49]
	AC-C	298	70.78	[50]
	Sulfonated Phenol-Formaldehyde Resin	298	103	[51]
	UiO-66-ND	298	39	[52]
	UiO-66-15	298	366	[52]
	Np-UiO-66	298	384	[63]
	G-SO_3H/Fe_3O_4	298	199.3	[54]
	Aluminium Pillared Clay	295	338	[55]
	SBRGBD	323	169.87	[56]
	HT-SDBS	301	40.5	[57]
	UiO-66	313	51.68	This work
	$Al_{0.3}Zr_{0.7}$-UiO-66	313	497.51	This work
MB	Cu-BTC	298	39.67	[58]
	NH_2-UiO-66	298	203.95	[59]
	UiO-66-P	298	91.1	[60]
	MIL-53(Al)-NH_2	308	208.3	[61]
	Fe_3O_4-COOH/HKUST-1	318	118.6	[62]
	TiO_2@GGH	303	198.61	[63]
	nFMBO	298	72.32	[64]
	UiO-66-NO_2	298	41.7	[65]
	CH–Mt/PANI base nanocomposite	298	111	[66]
	Cu-BTC@AG	303	282.47	[67]
	Porous Cellulose Bead	298	48.8	[68]
	Cal-Pal	298	57.47	[69]
	Ce(III)-doped UiO-66	298	145.3	[70]
	UiO-66	313	45.37	This work
	$Al_{0.3}Zr_{0.7}$-UiO-66	313	251.26	This work

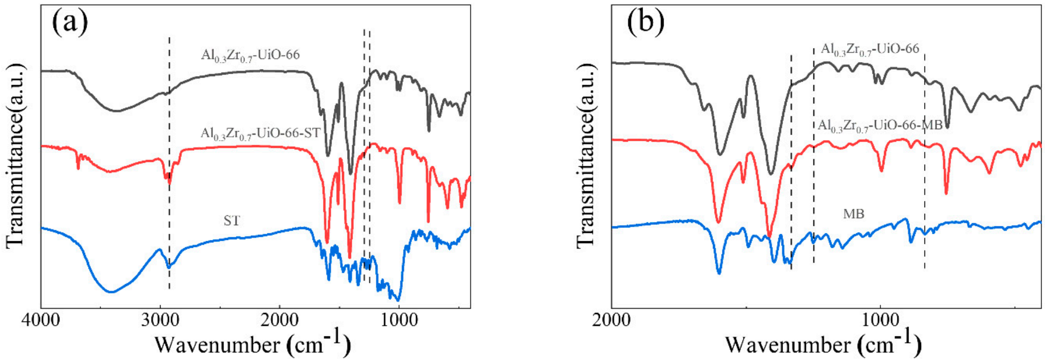

Figure S10. FTIR spectra of $Al_{0.3}Zr_{0.7}$-UiO-66 before and after adsorption.

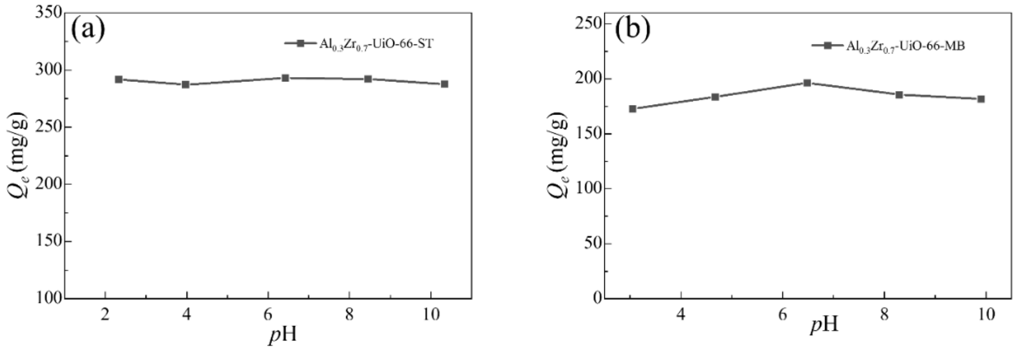

Figure S11. The adsorption amount of $Al_{0.3}Zr_{0.7}$-UiO-66 on (a) ST and (b) MB varied with *p*Hs.

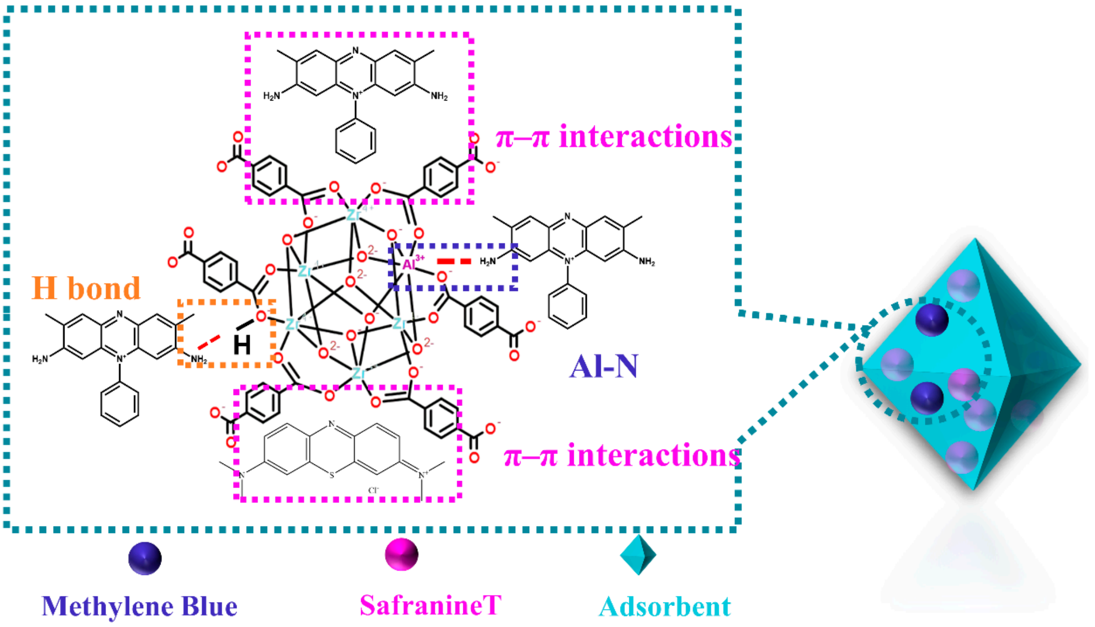

Figure S12. Schematic diagram of the proposed adsorption mechanism of two dyes ST and MB adsorption on Al-doped UiO-66.
